# A First Tetraplex Assay for the Simultaneous Quantification of Total α-Synuclein, Tau, β-Amyloid_42_ and DJ-1 in Human Cerebrospinal Fluid

**DOI:** 10.1371/journal.pone.0153564

**Published:** 2016-04-26

**Authors:** Niels Kruse, Michael G. Schlossmacher, Walter J. Schulz-Schaeffer, Eugeen Vanmechelen, Hugo Vanderstichele, Omar M. El-Agnaf, Brit Mollenhauer

**Affiliations:** 1 Department of Neuropathology, University Medical Center, Göttingen, Germany; 2 Program in Neuroscience and Division of Neurology, The Ottawa Hospital, University of Ottawa Brain and Mind Research Institute, Ottawa, ON, Canada; 3 R&D, ADx NeuroSciences, Gent, Belgium; 4 Neurological Disorders Center, Qatar Biomedical Research Institute, and College of Science and Engineering, Hamad Bin Khalifa University (HBKU), Education City, Qatar Foundation, Doha, Qatar; 5 Paracelsus-Elena-Klinik, Kassel, Germany and University Medical Center, Göttingen, Germany; Louisiana State University Health Sciences Center, UNITED STATES

## Abstract

The quantification of four distinct proteins (α-synuclein, β-amyloid_1-42_, DJ-1, and total tau) in cerebrospinal fluid (CSF) has been proposed as a laboratory-based platform for the diagnosis of Parkinson’s disease (PD) and Alzheimer’s disease (AD). While there is some clinical utility in measuring these markers individually, their usage in routine clinical testing remains challenging, in part due to substantial overlap of concentrations between healthy controls and diseased subjects. In contrast, measurement of different analytes in a single sample from individual patients in parallel appears to considerably improve the accuracy of AD or PD diagnosis. Here, we report the development and initial characterization of a first, electrochemiluminescence-based multiplex immunoassay for the simultaneous quantification of all four proteins (‘tetraplex’) in as little as 50 μl of CSF. In analytical performance experiments, we assessed its sensitivity, spike-recovery rate, parallelism and dilution linearity as well as the intra- and inter-assay variability. Using our in-house calibrators, we recorded a lower limit of detection for α-synuclein, β-amyloid_42_, DJ-1, and t-tau of 1.95, 1.24, 5.63, and 4.05 pg/ml, respectively. The corresponding, linear concentration range covered >3 orders of magnitude. In diluted CSF samples (up to 1:4), spike-recovery rates ranged from a low of 55% for β-amyloid_42_ to a high of 98% for DJ-1. Hillslopes ranged from 1.03 to 1.30, and inter-assay variability demonstrated very high reproducibility. Our newly established tetraplex assay represents a significant technical advance for fluid-based biomarker studies in neurodegenerative disorders allowing the simultaneous measurement of four pivotal makers in single CSF specimens. It provides exceptional sensitivity, accuracy and speed.

## Introduction

Reliable biomarkers are urgently needed for the diagnosis of Parkinson´s and other neurodegenerative diseases. Multiplexing several proteins to evaluate a pattern of analytes may aid in the (differential) diagnosis of several disease conditions [1–4]. The proteins α-synuclein (aSyn), β-amyloid_42_ (Aβ_42_), DJ-1 (PARK-7) and total tau (t-tau) are involved in the pathogenesis of neurodegenerative diseases, and have been proposed as biomarkers in cerebrospinal fluid (CSF) for a number of disease conditions with several hallmarks, such as amyloid plaques, neurofibrillary tangles, and Lewy bodies. Hong et al. [5] observed lower levels of CSF aSyn and DJ-1 in patients with Parkinson´s disease (PD) and Alzheimer´s disease (AD) than in control subjects. Shi et al. [1] and Mollenhauer et al. [6] found that aSyn and t-tau protein levels in the CSF are lower in three different aSyn-aggregation-related disorders, including PD, dementia with Lewy bodies (DLB), and multiple system atrophy (MSA) as compared to AD patients or neurological controls (NC). The combination of elevated t-tau protein (and phosphorylated tau protein) with reduced Aβ_42_ levels differentiates AD patients from controls with a sensitivity and a specificity of 80–90% [7]. The latter proteins are proposed as CSF biomarkers in research criteria for AD dementia and mild cognitive impairment (MCI) due to AD [8,9].

Multiplexing plays an important role in patient stratification to identify the presence of a specific disease type or pathology, and in the case of neurological disorders, to exclude other causes of dementia. Previous studies have shown that quantification of Aβ_42_, aSyn, and t-tau may help in the differential diagnosis of neurodegenerative diseases (as for example DLB), as they all reflect processes proximal to a specific neuropathology [10]. Although the pathophysiological process of neurodegeneration is related to the formation of oligomers or aggregates of aSyn, Aβ_42_ or tau, resulting in synaptic failures [11,12], the concentrations of the physiologically occurring monomeric, non-aggregated, form of the proteins are themselves altered during the disease process. Aβ_42_ concentrations are reduced in CSF of patients with AD and dementia with DLB [13]. T-tau levels in CSF of DLB patients are lower than in AD and higher than in PD patients [14,15]. The majority of biomarker studies in aSyn-aggregation-related disorders demonstrate reduced levels of aSyn in the CSF of patients with PD, DLB and MSA [1,16,17].

The aim of this study was to develop a sensitive, first-in-kind, immunoassay-based platform for the simultaneous measurement of aSyn, Aβ_42_, DJ-1 and t-tau in CSF, in order to provide a laboratory-based tool. This, to aid in the objective, reliable and reproducible differential diagnosis of distinct neurodegenerative illnesses, to estimate disease progression, and in the future, to monitor the utility and efficacy of new compounds in therapeutic intervention studies. The major advantage of a multiplex assay format is that it reduces costs and processing time. The entire assay may be performed within less than four hours. In addition, this format may also help increase diagnostic accuracy by measuring four analytes in a single sample wihout the need to run multiple assay plates. Therfore it will also maximize the amount and value of information collected from very small sample volumes [18]. A potential disadvantage of multiplex assay formats is any non-specific interaction between antibodies and non-cognate analytes in the reaction mix. Such potential limitations have to be addressed experimentally and, if present, solved operationally. The purpose of our study was to improve conditions and output for investigators when processing human CSF samples for diagnostic purposes (regarding time, convenience, sensitivity, specificity and value of information). Therefore, we built and systematically explored a (first) tetraplex platform for four relevant analytes linked to neurodegenerative disorders.

## Methods

All measurements were performed on the MSD Sector Imager 6000. We used Multi-Array Standard Plates for the development of singleplex assays from Meso Scale Discovery^™^ (MSD, Gaithersburg, MD, USA); recombinant proteins which were used as calibrators were from [19] (aSyn), Covance (Münster, Germany) (DJ-1), rPeptide (Bogart, GA, USA) (tau protein 441), and MSD (Aβ_42_).

### Development of singleplex and multiplex assays

First, we developed single analyte assays on the platform. The singleplex assay procedures and optimization of the assay formats were performed as described previously [20]. In a comparable approach, we established sensitive and reproducible singleplex assays for three analytes with the following combination of antibodies: aSyn 3.0 μg/ml rabbit antibody clone 12.1 (kindly provided by Liyu Wu, Epitomics, Burlingame, CA, USA, now available through abcam, Cambridge, UK, as MJFR1) [21] as capture antibody and 1.0 μg/ml Syn-1 (BD Biosciences, Heidelberg, Germany) as detection antibody; human DJ-1 antibody AF3995 (R&D Systems, Wiesbaden, Germany) both as capture and as detection antibody at concentrations of 0.15 and 0.1 μg/ml, respectively. T-tau protein was quantified using a combination of 1.0 μg/ml antibody ADx215 for capture and ADx201 (both from ADx Neurosciences, Gent, Belgium) for detection; for the quantification of Aβ_42_, the assay, as developed by MSD [22] was incorporated into the tetraplex assay.

Detection antibodies were tagged with [ruthenium(II) tris-bipyridine-(4-methylsulfonate) NHS ester] (SULFO-TAG) according to the manufacturer’s instructions, except for ADx201, which was biotinylated and used in combination with 0.5 μg/ml streptavidin-SULFO-TAG (MSD). Ruthenium tagged antibodies are quantified using electrochemiluminescence (ECL) technology. Unspecific binding of AF3995 and ADx201 was quenched by adding 0.1% mouse (Rockland Immunochemicals, Limerick, PA, USA) and goat immunoglobulin (Equitech-Bio, Kerrville, TX, USA) to the detection antibody solution.

Calibration curves were prepared with recombinant proteins dissolved in 1% BSA/0.05% PBS-Tween20 (PBS-T) covering a concentration range from 6.1 to 25.000 pg/ml (aSyn, DJ-1, and t-tau protein) or 0.73 to 3000 pg/ml (Aβ_42_). PBS-T was used as blank control. Concentrations as mentioned on the data sheet were used for the study.

The antibodies for the multiplex assay plates were spotted in the facilities of MSD according to their standard operation procedures. The following coating conditions were applied: 0.5x MSD coating concentration of AF3995, 1x coating concentration of MJF1, ADx215 and MSD´s Aβ_42_ capture antibody. Quality control of the coating efficiency was carried out by MSD. For the multiplex assays, we generally blocked and diluted calibrators or samples with Diluent 35 (MSD) instead of 1% BSA/PBS-T. Calibrators and samples were applied to the system at 25 μl for 1h in singleplex and 50 μl for 2h in multiplex assays.

### Partial qualification of the multiplex assays

For the qualification experiments, randomly selected CSF samples from controls of the “Kassel cohort” were used. Details on sample processing and diagnostic criteria were published recently [6].

### Performance tests

#### Spike-recovery rates

Initial experiments to determine the recovery rates of spiked recombinant proteins in undiluted CSF samples indicated matrix interference for each analyte, resulting in low relative values (29.2%, 20.1%, 46.5, and 86.0% for aSyn, Aβ_42_, DJ-1 and t-tau protein, respectively). Therefore, CSF samples were diluted up to 1:4 in all further experiments. First, recovery rates were determined in CSF, prepared by pooling eight individual CSF samples that were then diluted 1:4 with Diluent 35. In this experiment, calibrator proteins were spiked individually at a final concentration of 250, 500, and 1,000 pg/ml for Aβ_42_ and t-tau protein or 625, 1,250, and 2,500 pg/ml for aSyn and DJ-1. In parallel, all four proteins were also spiked as an entire set at the same concentrations into our pooled CSF specimen. Spiking was performed by adding one volume of individual or combined calibrators to nine volumes of CSF. Endogenous levels of protein were determined by spiking one volume Diluent 35 without calibrators to nine volumes of CSF. The latter is important in order to account for the dilution effect by the addition of the diluent to the CSF. In addition, nine volumes of Diluent 35 were spiked with one volume of the calibrators to verify the exact concentration of each analyte that was spiked into the samples. Finally, we determined spike-recovery rates in individual CSF samples with a procedure identical to that described above, but using only pooled protein calibrators. Addition of proteins was done immediately prior to analysis without additional freezing of samples.

#### Dilution linearity and parallelism

Dilution linearity was determined by adding approximately 100-times the endogenous protein concentration of aSyn, DJ-1 and t-tau protein and 25-times the endogenous Aβ_42_ concentration to three individual CSF samples. The final protein concentrations were 20 ng/ml aSyn, 7 ng/ml Aβ_42_, 320 ng/ml DJ-1, and 28 ng/ml t-tau protein. Spiked CSF samples were then serially diluted two-fold in Diluent 35 for analysis.

Parallelism of protein quantification was verified by preparing serial two-fold dilutions of pooled CSF (see above) and three individual CSF samples for analysis. Values were re-calculated taking into account the dilution factor as described [23].

Raw data were analyzed using Workbench 3.0 software (MSD).

Our study was carried out in accordance with the Declaration of Helsinki and with the informed written consent of all patients or, in the case of cognitive impairment, their next of kin. Our study was approved by the ethics committees of the University of Göttingen and the Landesärztekammer Hessen (Germany).

#### ELISA assays

Quantification of Aβ_42_, t-tau and aSyn were performed according to instructions and protocols provided by the supplier (INNOTEST ELISA Fujirebio Europe, Ghent, Belgium and BioLegend, San Diego, USA).

#### Statistical analysis

Results with CVs <20% for replicate determinations of analyte concentrations were considered reliable according to published guidelines [23–25]. Group comparison was performed using the t-test. Statistical analysis was performed using GraphPad Prism Version 5.01 software (San Diego, CA, USA). All other calculations were performed using Microsoft Office Excel 2003.

## Results

### General assay performance

We first optimized an established, sandwich-type aSyn ELISA [26,6] and developed new single analyte assays for quantification of DJ-1 and t-tau protein in CSF. All assays were optimized with respect to analytical sensitivity and reproducibility, as described previously [20]. Acceptance criteria for qualification of the singleplex assays included a (1) large dynamic range comprising more than 3 orders of magnitude, (2) acceptable dilution linearity for CSF to avoid matrix-effects, and (3) good correlation between signal intensity and protein concentration.

The general characteristics of tetraplex assay performance are summarized in Table 1. Correlation of increase in signal intensity and protein concentration (referred to as Hillslopes) ranged from 1.03 to 1.30 with very low variability.

**Table 1:** Analytical sensitivity and lower limit of detection for tetraplex assay.

Hillslope	Mean	SD	%CV
aSyn	1.2	0.02	1.5
Aβ_42_	1.3	0.03	2.7
DJ-1	1.2	0.05	4.6
Tau	1.0	0.04	3.8
LLOD (pg/ml)	Mean	SD	%CV
aSyn	2.0	1.7	87.2
Aβ_42_	1.2	0.9	71.8
DJ-1	5.6	4.6	82.3
Tau	4.1	3.3	81.5

Data were calculated from seven individual experiments.

### Reproducibility of calibration curves

**Fig 1:**
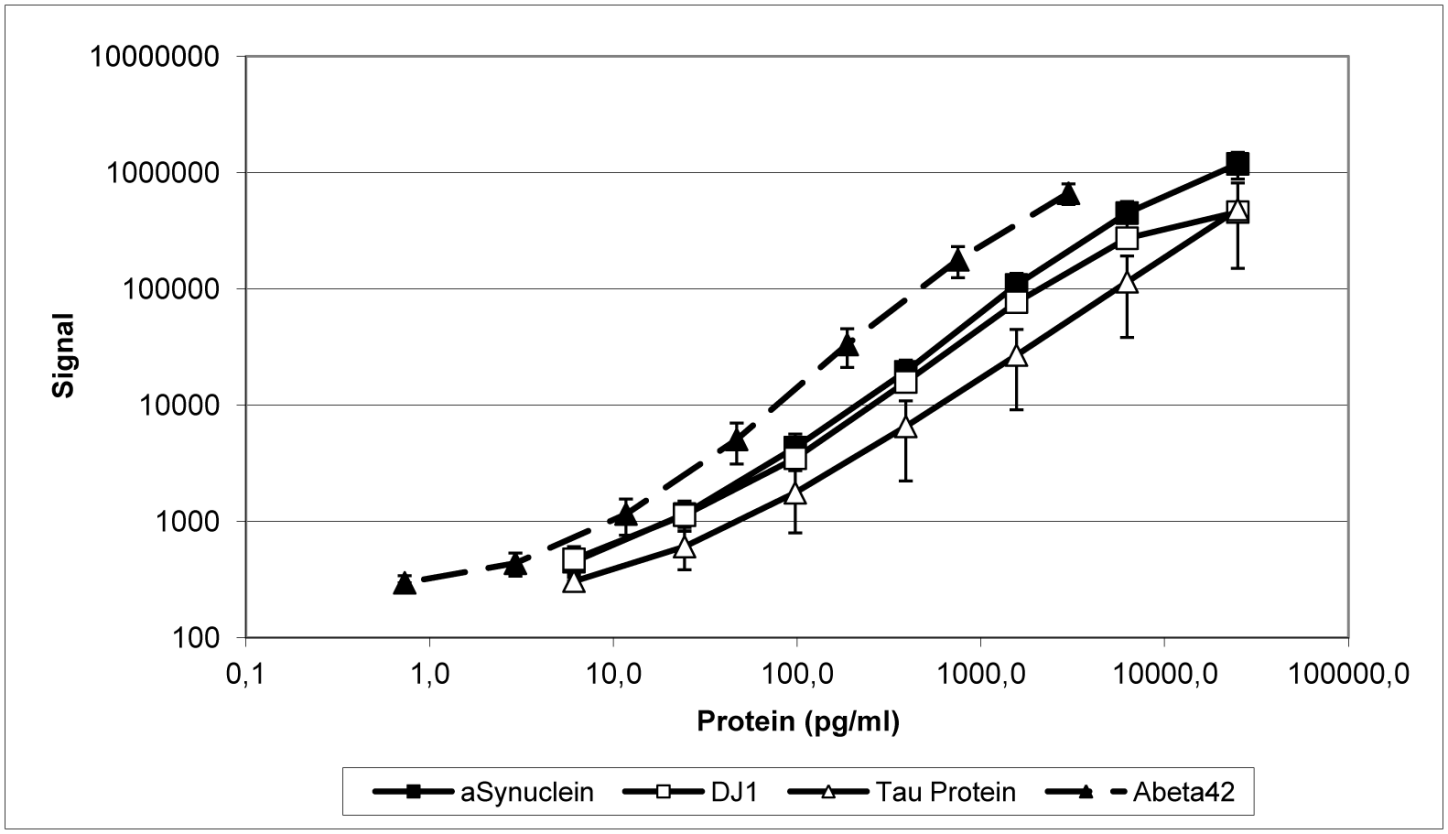
Calibration curves of recombinant proteins are highly reproducible. Normalized mean values (± standard deviation) from seven independent experiments performed with multiplex assay plates are presented.

The calibrators were prepared in serial four-fold dilutions ranging from 25,000–0 pg/ml (aSyn, DJ-1 and t-tau protein) or 3,000–0 pg/ml (Aβ_42_). Fig 1 shows the mean results from seven independent experiments (see also S1 Table). Data are presented as the percentage of signals at maximum concentration. Variability in signal intensity was low, with CV below 20%, except for the lowest concentration of DJ-1 and some t-tau concentrations. Comparable results were obtained when back-calculated calibrator concentrations were used for analysis.

### Sensitivity of assay

The lower limits of detection (LLOD), as determined by Workbench 3.0 software, varied between 1.24 pg/ml and 5.63 pg/ml and therefore confirmed the high sensitivity of each component of the assay (Table 1). For LLODs, relatively large standard deviations (SD) and coefficients of variance (%CV) were observed, which was to be expected as ELISA usually have considerable variability at the upper and lower ends of their calibration curves, especially within transitions from a linear phase to the plateau phase.

### Potential protein-protein interactions

**Fig 2:**
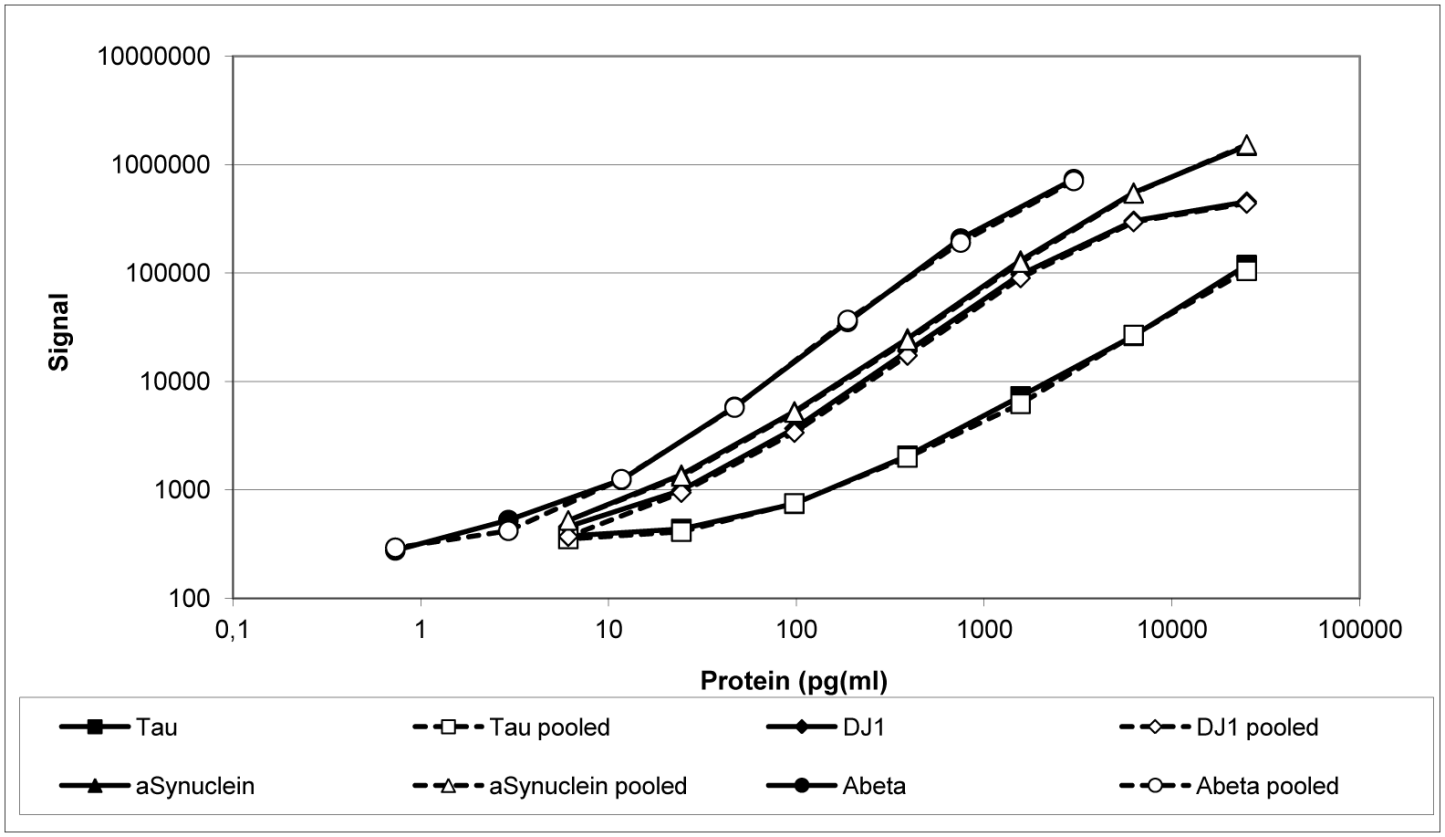
Highly comparable performance in quantification of pooled versus individual recombinant proteins on multiplex assay plates. Individual as well as pooled recombinant calibrators were serially diluted by a factor of three for analysis. Signal intensities were comparable under both conditions.

Potential interactions in our assay format with other proteins present in the biological fluid specimen were analyzed in two different ways. First, calibration curves of pooled and individual proteins were compared. Calibration curves were compared within the same assay run. As shown in Fig 2 and S2 Table, no differences were found between signal intensities or the slopes of calibration curves derived using either individual proteins or pooled calibrators.

In a second experiment, the four recombinant calibrators were combined in all possible combinations at 25,000 pg/ml (aSyn, DJ-1 and t-tau protein) and 3,000 pg/ml (Aβ_42_), respectively (Table 2 and S3 Table). According to the calibration curves, the concentrations of the proteins measured separately nearly always matched the expected concentrations with less than 20% deviation. In these assays, both aSyn and t-tau protein concentrations, regardless of relative amounts, were within 20% of the expected value. In rare cases, Aβ_42_ and DJ-1 quantification generated deviations by >20% of the expected value. Together these data showed the absence of strong protein-protein interactions under our assay conditions, i.e., when the CSF were dissolved in 1% BSA solution.

**Table 2:** Analysis of protein interactions.

	Proteins in assay				Concentration determined (pg/ml)				Percentage of individual protein concentration			
Assay	aSyn	Aβ_42_	DJ-1	tau	aSyn	Aβ_42_	DJ-1	tau	aSyn	Aβ_42_	DJ-1	tau
1	x	x	X	x	25246	4899	27505	28162	102.5	139.3	119.1	98.5
2	x	x	x		26429	3583	28378		107.3	101.9	124.4	
3	x	x		x	25432	3616		28264	103.3	102.8		98.9
4	x		x	x	24464		27952	27343	99.4		121.0	95.7
5		x	x	x		3520	25497	26847		100.1	110.4	93.9
6	x	x			25720	3480			104.5	99.0		
7	x		x		26065		29055		105.9		125.8	
8	x			x	26470			31090	107.5			108.8
9		x	x			3632	25172			103.3	109.0	
10		x		x		3560		27912		101.2		97.7
11			x	x			21265	26936			92.1	94.3
12	x				24625				100.0			
13		x				3517				100.0		
14			x				23097				100.0	
15				x				28581				100.0

Recombinant proteins were combined in 15 possible combinations (left panel) for quantification. Concentrations for the different proteins determined from pooled sample assays are summarized (middle panel) and normalized for the concentration of the individual protein (right panel). Note the infrequent values with deviations of more than 20% from the expected values.

### Dilution linearity

**Fig 3:**
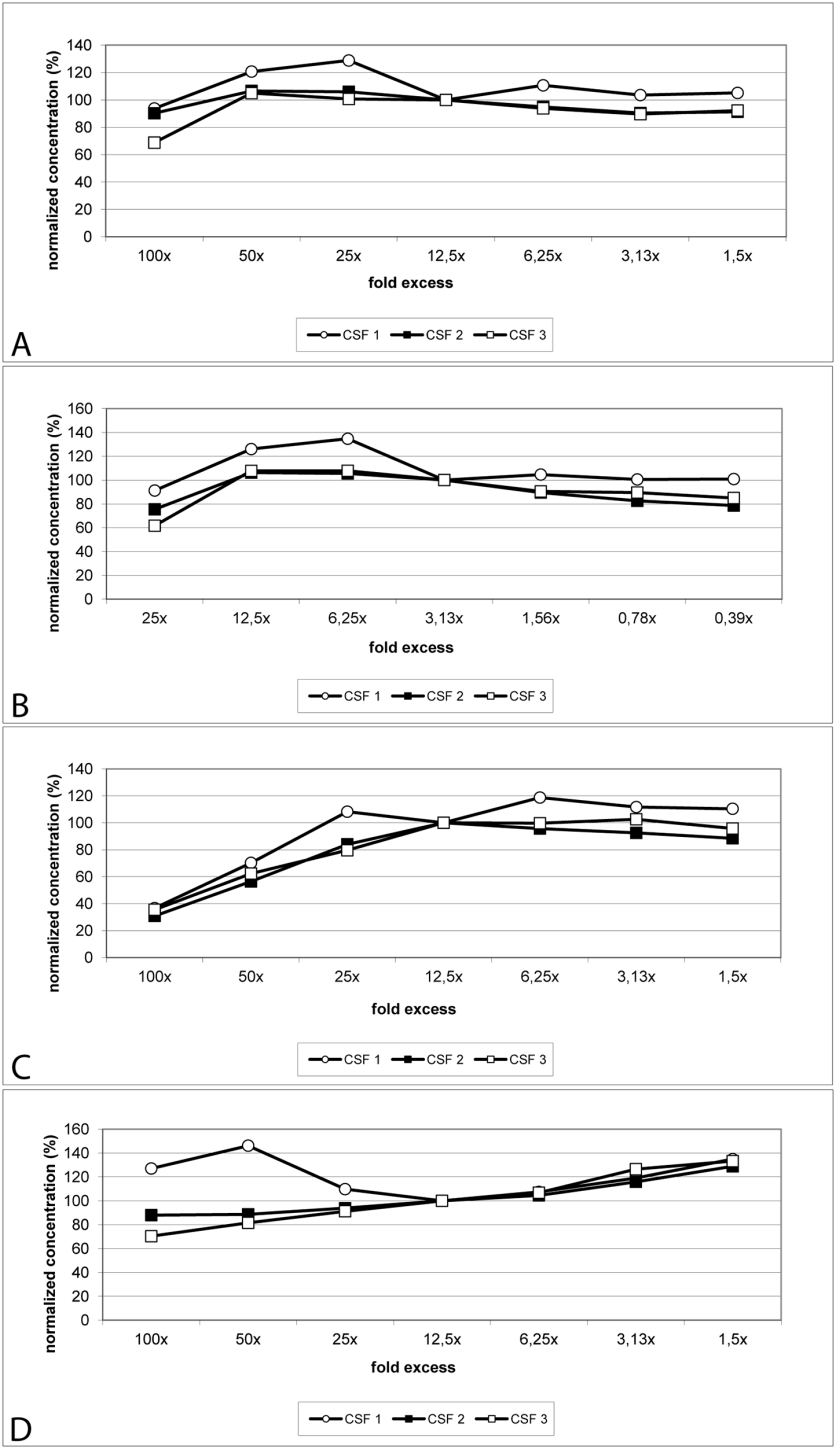
Linearity testing for serial dilution of proteins spiked into CSF samples. Three CSF samples were spiked with approximately 100 times the endogenous concentration of aSyn, DJ-1 and t-tau protein as well as 25 times of endogenous Aβ_42_ concentration. CSF samples were serially diluted by a factor of two for analysis. Protein concentrations were normalized for the fourth dilution. Relative protein concentrations are presented. A: aSyn, B: Aβ_42_, C: DJ-1, D: t-tau protein.

Dilution linearity was analyzed using serial two-fold dilutions of three individual CSF samples spiked with an approximately 100-fold endogenous concentration of aSyn, DJ-1, and t-tau protein as well as a 25-fold endogenous concentration of Aβ_42_. Results normalized for the third dilution (i.e. a 12.5- or 3.13-fold excess of exogenous protein concentration spiked into CSF) are presented in Fig 3 and S4 Table. At protein concentrations exceeding a 12.5-fold endogenous concentration of aSyn, DJ-1 and t-tau protein and a 3.13-fold endogenous Aβ_42_ concentration, normalized protein concentrations differed by more than 20% from the reference concentration. At higher dilutions normalized concentrations were comparable to the reference values until, in the case of t-tau protein, the concentration approached the detection limit and therefore protein quantification became more inconsistent.

### Parallelism

**Fig 4:**
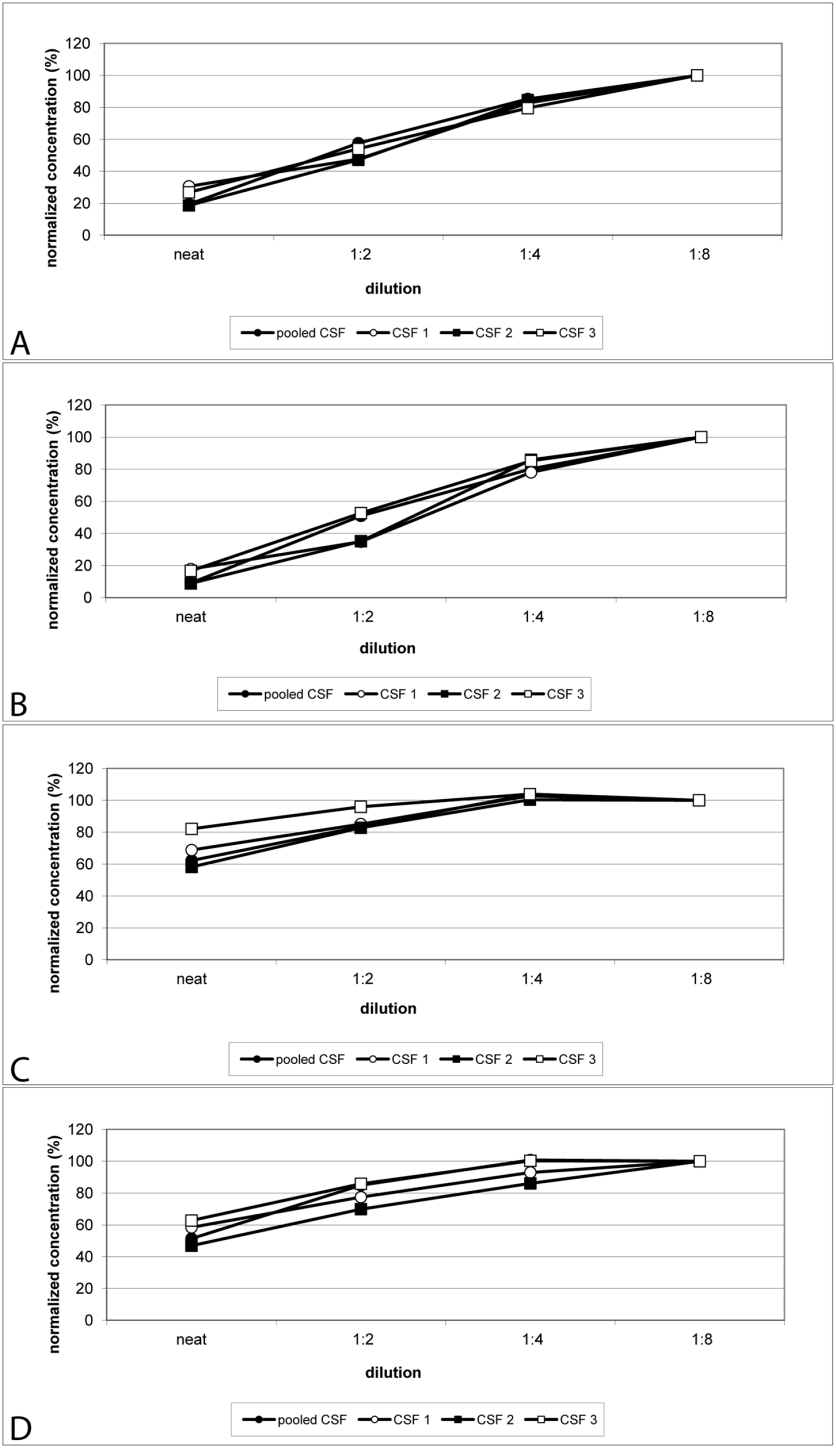
Parallelism of analyte quantification in serially diluted CSF samples. Pooled CSF and three individual CSF samples were serially diluted by a factor of two for analysis. Protein concentrations were normalized for the 1:8 dilutions. A: aSyn, B: Aβ_42_, C: DJ-1, D: t-tau protein.

Parallelism was analyzed using serial two-fold dilutions of CSF, and using undiluted CSF as the highest concentration. Results normalized for concentrations in CSF diluted at 1:8 are presented in Fig 4 and S5 Table. Results of normalized protein concentrations became more comparable with increasing dilution. While protein concentrations, as measured in undiluted CSF and 1:2 diluted CSF specimens, were significantly lower than the reference concentration, most of the values determined at 1:4 were comparable to the reference data. For aSyn and Aβ_42_, one out of four samples measured at 1:4 dilution deviated by slightly more than 20% from the reference value measured at 1:8 dilution (20.5 and 22%, respectively). These results point to matrix effects interfering with the protein quantification at higher CSF concentration.

### Accuracy rates (recovery of spiked proteins)

Initial experiments using undiluted CSF samples to analyze recovery rates for spiked proteins indicated that matrix effects significantly inhibited protein quantification. We therefore used a pool of eight distinct CSF samples diluted at 1:4 with Diluent 35 and spiked with individual or pooled calibrators. Recovery rates improved considerably following CSF dilution: Using three different spike concentrations of each protein, mean recovery rates for individually spiked proteins were determined as 70.5 ± 2.6% for aSyn, 73.0 ± 4.3% for Aβ_42_, 105.7 ± 3.0% for DJ-1, and 119.5 ± 9.7% for t-tau protein (Table 3A). Similarly, slightly lower recovery rates were found when pooled proteins were spiked into pooled CSF, where recovery rates were 60.7 ± 9.7% for aSyn, 58.2 ± 13.3% for Aβ_42_, 98.6 ± 1.8% for DJ-1, and 106.6 ± 9.0% for t-tau protein (Table 3A). This experiment was repeated with four individual CSF samples using pooled proteins. Under these conditions recovery rates were at 64.3 ± 3.6%, 55.5 ± 3.7%, 98.0 ± 5.4, and 95.7 ± 7.1% for aSyn, Aβ_42_, DJ-1 and t-tau protein, respectively (Table 3B, see also S6 Table).

**Table 3:** 

A. %Recovery rates of spiked protein calibrators into pooled CSF								
	aSyn		Aβ_42_		DJ-1		tau protein	
spike concentration	pooled calibrators	individual calibrators	pooled calibrators	individual calibrators	pooled calibrators	individual calibrators	pooled calibrators	individual calibrators
High	61.1	69.6	55.2	69.8	97.3	102.8	99.5	115.1
Medium	50.8	73.2	46.7	72.2	97.8	108.7	103.5	130.6
Low	70.2	68.5	72.8	77.8	100.7	105.4	116.7	112.9
Mean	60.7	70.5	58.2	73.0	98.6	105.7	116.7	112.9
SD	9.7	2.6	13.3	4.3	1.8	3.0	9.0	9.7
B. % Recovery rates of spiked calibrators into four individual CSF samples								
	aSyn		Aβ_42_		DJ-1		tau protein	
spike concentration	mean recovery (±SD)							
high	63.1 (19.8)		55.6 (15.6)		91.9 (5.19		103.5 (14.6)	
medium	68.3 (21.8)		59.1.(15.0)		102.2 (2.6)		94.0 (11.5)	
low	61.4 (16.9)		51,7 (13.4)		99,8 (3.3)		89,6 (7.2)	
mean	64.3		55.5		98.0		95.7	
SD	3.6		3.7		5.4		7.1	

**Fig 5:**
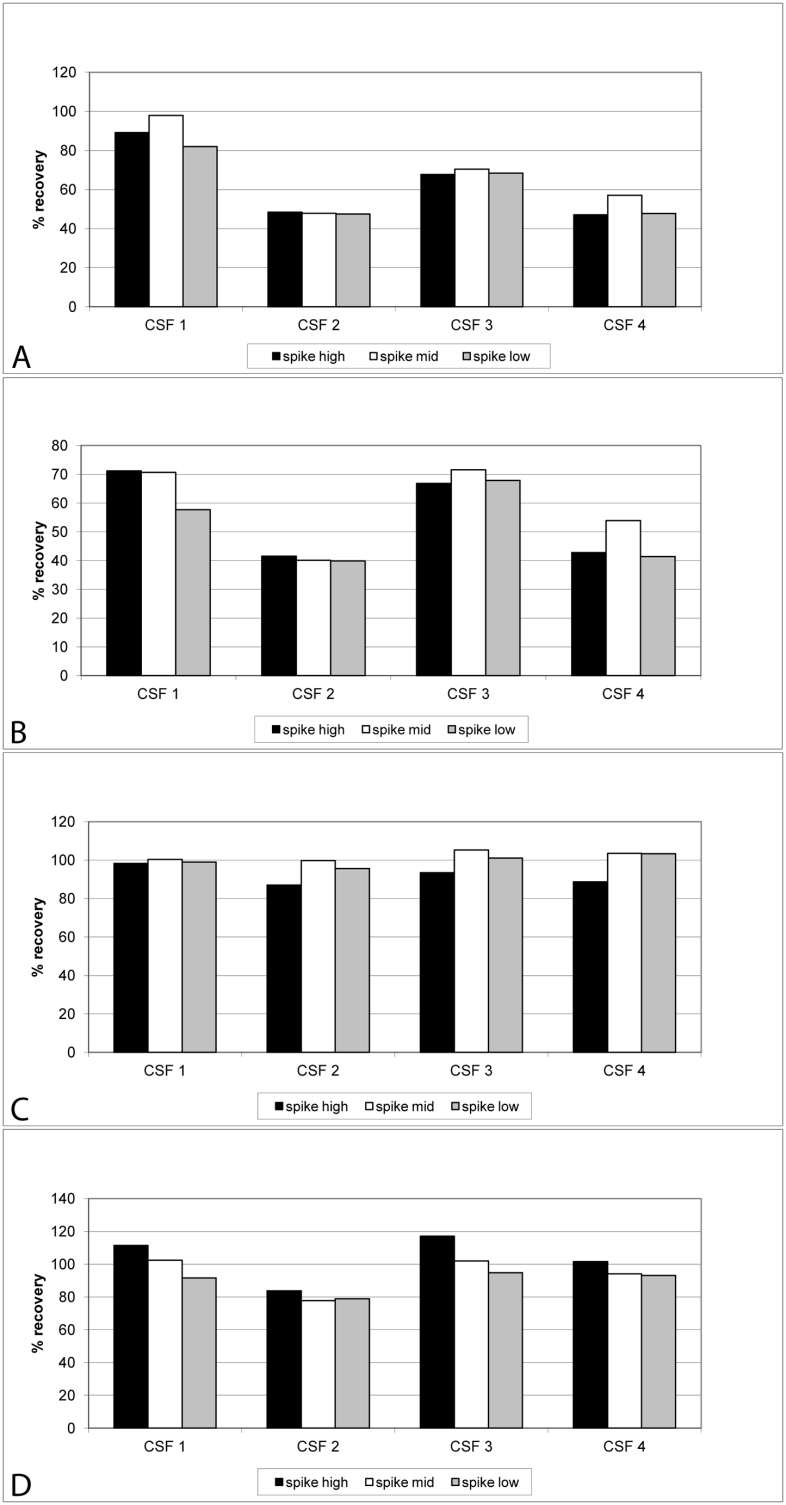
Recovery rates of spiked calibrators into individual CSF samples. Recombinant calibrators were spiked at three different concentrations into four CSF samples. Concentrations of spike solutions and endogenous protein concentrations were determined in parallel. Recovery rates were calculated taking into account both the endogenous CSF concentration and the protein concentration of the spike solutions. A: aSyn, B: Aβ_42_, C: DJ-1, D: t-tau protein.

Except for DJ-1, relatively high standard deviations of recovery rates were noted with individual CSF samples (Fig 5). We observed that in two out of four CSF samples (CSF2 and CSF4) recovery rates were lower than for CSF1 and CSF3. This implies that although CSF samples were diluted by a factor of four to reduce matrix effects, these were still observable, albeit much less so compared to undiluted CSF samples. One might consider in the future a higher dilution factor for such samples, as long as the working range for analyzing the samples is not negativelyaffected.

#### Intra- and inter-assay variability

Intra-assay reproducibility was determined by measuring CSF samples in duplicate. In total, 36 undiluted CSF samples were measured and 192 were measured at 1:4 dilution. Most samples showed CVs below 20%. For undiluted CSF samples, CV values above 20% were found in 3 cases for aSyn (range: 23.9–35.4%), 5 cases for Aβ_42_ (range: 22.9–52.9%), 2 cases for DJ-1 (range: 23.3–30.5%) and 3 cases for t-tau protein (range: 21.9–30.0%). For diluted CSF samples, CVs above 20% were found in ten cases for aSyn (range: 20.4–28.3%), 16 cases for Aβ_42_ (range: 22.5–46.3%), one case for DJ-1 (CV: 28.7%) and 27 cases for t-tau protein (range: 20.5–141.1%).

Inter-assay variability was determined by measuring 40 CSF samples at 1:4 dilution in two independent experiments. Two CSF samples showed CVs above 20% for quantification of Aβ_42_ (23.2 and 27.5%). T-tau protein quantification yielded CVs above 20% in 20 cases. With the exception of four CSF samples that exhibited relatively high t-tau protein concentrations, the other 16 CSF samples had t-tau protein concentrations at or near the lower limit of detection (i.e. below 20 pg/ml). Quantification of aSyn and DJ-1 concentrations always gave CVs below 20%.

To compare performances between our tetraplex prototype and individual commercial platforms in a larger sample set, we chose a subset of 83 CSF specimens with enough remaining volume for quantification in multiple different assays for values of Aβ_42_, t-tau and aSyn. As expected, the results revealed a high correlation between them for all three proteins tested (R^2^ for aSyn: 0.767, for Aβ_42_: 0.559, for Tau: 0.725) (S1 Fig).

### Comparison of quantification results in singleplex and multiplex assays

**Fig 6:**
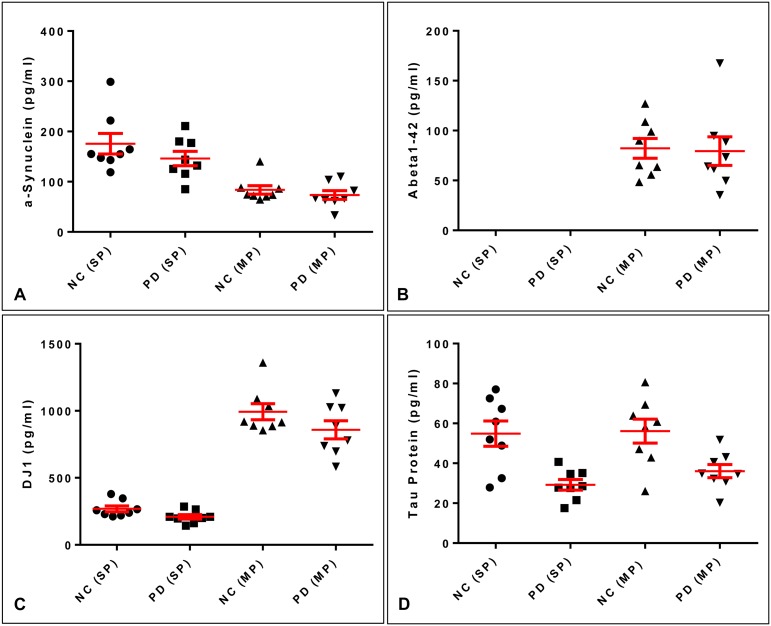
Quantification of CSF samples in singleplex versus multiplex assays. Eight CSF samples from neurological controls and patients with PD were analyzed both in singleplex (except for Aβ_42_) and (SP) multiplex assays (MP). Results were obtained from non-diluted samples.

For a first pilot set of CSF samples submitted for diagnostic purposes in the differential diagnosis of motoric impairment, we analyzed eight patients with normal pressure hydrocephalus (NC) without evidence of typical PD and eight patients with idiopathic PD. Twenty five μl of CSF were measured undiluted both in our singleplex assays for aSyn, DJ-1 and t-tau protein and by tetraplex assay (Fig 6 and S7 Table). Overall concentrations of aSyn were lower in the tetraplex assay than by singleplex assay. The reverse was true for DJ-1. The t-tau protein results were not statistically different between singleplex and tetraplex assays. Samples from PD patients showed lower protein levels of aSyn, DJ-1 and t-tau protein as compared to NC patients on the multi- and singleplex assay. This difference was statistically different for DJ-1 and t-tau protein in singleplex quantifications and also for t-tau protein in multiplex quantifications (Table 4). Protein concentrations determined in both assay formats were highly correlated (R^2^ for aSyn: 0.504, for DJ-1: 0.791, for Tau: 0.726) (S2 Fig).

**Table 4:** P-value summary for comparison of singleplex and multiplex quantification.

p-value	aSyn	Aβ_42_	DJ-1	tau protein
singleplex NC vs. singleplex PD	0.2606	n.d.	0.0458	0.0023
Multiplex NC vs. multiplex PD	0.4172	0.8753	0.1548	0.0112
singleplex NC vs. multiplex NC	0.001	n.d.	<0.0001	0.8899
singleplex PD vs. multiplex PD	0.0007	n.d.	<0.0001	0.1281

## Discussion

In this study we describe the first generation of a tetraplex immunoassay that simultaneously quantifies aSyn, DJ-1, Aβ_42_ and t-tau protein in CSF on an ECL-platform; furthermore, its performance was substantiated according to the most stringent criteria, such as those published in internationally recommended guidelines for new assay validation [23–25]. With the recent increasing number of multiplex assays developed for multiple disciplines in clinical management and preclinical studies, researchers and clinicians have the opportunity to measure multiple biomolecules in parallel. Application of ECL-detection moreover allows a sensitive quantification of up to ten analytes in parallel.

Singleplex immunoassays are suitable to measure individual protein concentrations without the problem of potential interference of antibodies. However, multiple assays need to be performed to quantify several proteins. Furthermore traditional ELISAs often require sample incubation over night.

The major advantage of a multiplex assay format is that it reduces time, cost and processing time. The whole assay may be performed within less than four hours. In addition this format may help to increases diagnostic accuracy by measuring four analytes in a single sample without the need to run multiple assay plates, which can introduce variability due to multiple handling steps of the same stock specimen. Therefore it is desirable to derive considerably more information from minimal sample volumes [18]. A disadvantage of multiplex assay formats is the occurrence of non-specific interaction of the antibodies and analytes used in the reaction. These have to be addressed experimentally, which we did in the case of our chosen four analytes.

The ECL technology is widely used in different research areas [27,28]. Most studies used commercially available assays, which were usually not extensively validated. Detailed studies on validation of custom-made ECL assays have been published recently [29,30].

Our assay development efforts were aimed at multiplexing four proteins associated with Parkinson´s or Alzheimer´s disease pathology, i.e. aSyn, DJ-1, t-tau protein and Aβ_42_, in order to improve diagnostic accuracy by identifying PD pathology or by excluding AD pathology.

The general performance of our established multiplex assay as indicated by the Hillslopes and LLODs showed high sensitivity for each of the four proteins of interest. Hillslopes define the correlation of increases in concentration and signal intensity. A sensitive assay should have very close correlation between these two metrics. An ideal Hillslope of 1 demonstrates that a two-fold or ten-fold increase in concentration corresponds to a two-fold or ten-fold increase in signal intensity, respectively. In our experiments we observed mean Hillslopes of 1.30 for Aβ_42_. These data show that in the case of Aβ_42_ a ten-fold increase in protein concentration corresponds to a 13-fold increase in signal intensity. In other words, a 13-fold increase in signal intensity is required to measure a ten-fold increase in protein concentration. For the other proteins the correlation was much better. Mean Hillslopes ranged from 1.03 to 1.15. In addition, LLODs for all proteins were far lower than the lowest concentrations measured in our clinical samples (1.24–5.63 pg/ml).

### Reproducibility of calibration curves

The calibration curves were found to be highly reproducible. The mean signal intensities obtained at all concentrations performed in duplicate in seven experiments gave CV values of less than 20% in most cases, indicating that quantification over the entire concentration range is reliable [23–25].

### Intra- and inter-assay variability

Both in undiluted and in diluted CSF we found that most of the duplicate determinations had CVs below 20%, except for t-tau protein. Signal readings for t-tau protein in most cases was detected at the lower end of the calibration curve, where slight changes in signal reading have a strong impact on concentration assignment. This is very evident when diluted samples were analyzed. In this case the proportion of t-tau protein measurements with high CVs increased and therefore became less reliable. Quantification of the other proteins was comparable in two experiments. These results clearly demonstrate that finding a single dilution factor that permits the measurement of multiple proteins in a given sample may be difficult, as has been reported [3].

### Potential interaction of proteins

All proteins of interest in this study tend to form either homo- or hetero-oligomers or to promote aggregation of other proteins [31–33]. We therefore analyzed whether significant protein interactions occurred by comparing 1) the performance of calibration curves of individual proteins with those of pooled calibrators and 2) co-incubation of all proteins of interest at very high concentrations in all possible combinations. Neither the comparison of curves for individual or pooled calibrators nor mixtures of proteins at high concentration give any hint of protein aggregation.

### Accuracy (recovery)

Recovery rates determined in several experiments showed that these were higher than 50% for all proteins and nearly 100% for t-tau protein and DJ-1. They were slightly lower for aSyn and Aβ_42_. These results clearly show that the combinations of antibodies used in this study are suitable for quantification of the proteins in a multiplex format. We observed that using individual CSF samples led to variable recovery rates. This may be attributed to matrix effects which can be reduced by further diluting the CSF. Further reduction of CSF concentration will also reduce t-tau protein concentrations to levels below the limit of detection, at least in our cohort. The analytical sensitivity of the t-tau assay can be improved by combining it with other monoclonal antibodies or modifying the method of labelling the detector antibody. Nevertheless, this multiplex assay format may be suitable for analysing t-tau protein concentrations in other neurodegenerative diseases that are accompanied by significant cell destruction (for example Alzheimer’s disease and Creutzfeldt-Jakob disease).

Our multiplex assay does not necessarily preclude problems that arise with immunoassay performance in general, such as lab-to-lab variability, lot-to-lot variability, lack of certified reference material, etc. [34,35]. However, increasing implementation of automated systems promises to limit errors generally associated with the performance of ELISAs and comparable assays.

### Comparison of test results between singleplex and multiplex assays

We compared the quantification results of our multiplex assay with those of singleplex assays in a small cross-sectional study. In these experiments we found that the general trend was comparable between both assay formats, in that the CSF samples from the PD patients had lower concentrations of aSyn, DJ-1 and t-tau protein than those from the patients with normal-pressure hydrocephalus used as clinical controls. These results are consistent with other studies investigating CSF levels of these proteins in PD patients and healthy controls [1,5,36]. Interestingly, aSyn revealed lower concentrations in the multiplex assay than in singleplex assays, while the reverse was true for DJ-1. T-tau protein levels were determined to be comparable in both assays. The reason for these discrepancies is currently not known and will be investigated in further experiments. The most obvious explanation is that antibody concentrations differed between the two assays. Compared to custom-made singleplex assays, much higher antibody concentrations are used to coat spots on multiassay plates. This fact in combination with spatial reading of the comparably small spots on multiassay plates might be responsible for this observation. In addition, we found that Hillslopes differed between assay formats, further contributing to differences in concentration calculations. Results from the analysis of parallelism clearly demonstrated that with increasing CSF dilution the relative analyte concentration increased. Matrix effects seem to interfere with quantification of our proteins of interest, in particular for aSyn and Aβ_42_. We speculated that similar observations will be made when diluted samples from PD patients are compared to neurological controls. Currently we are not able to definitely state whether the effect of diluting CSF will lead to a more comparable concentration assignment between the two different assay formats. The answer would require that in the case of DJ1 sample dilution must have a very strong effect in the singleplex assay in order get comparable results to the multiplex assay while for α-Synuclein the effect should be higher in the multiplex assay. Further experiments are needed to address this issue.

We found that for defined calibrator concentrations much higher ECL signals were measured on multiplex than on singleplex plates. This was also seen in the samples, although not to the same extent. ECL signals for t-tau protein calibrators and samples were comparably increased on multiplex plates (~3.5-fold). With aSyn, the ECL signals for calibrators and samples on multiplex plates increased by factors of four and two, respectively, while the calibrator readings for DJ-1 increased ~20-fold and sample readings >70-fold on multiplex plates. Therefore DJ-1 concentrations were assigned much higher levels on multiplex plates and aSyn concentrations to lower levels as compared to singleplex plates.

## Conclusions

We demonstrate the generation and careful characterization of a tetraplex assay for the simultaneous quantification of PD- and AD-associated proteins in as little as 50 μl of CSF volume. The assay is easy to perform, requires less hands-on time as compared to conventional ELISAs and saves precious clinical material. Further experiments are required to validate and apply the assay further, such as to demonstrate its usefulness in routine laboratory practice processing larger numbers of samples than shown here. Major advantages of the tetraplex assay are (1) its superior sensitivity, (2) the broad dynamic range, (3) its high degree of reproducibility; and importantly, (4) the one-step readout of multiple analytes in the same specimen. We are aware that our assay also needs validation by independent groups (e.g. round robin trials [35]). Due to the complexity of multiplexing there is a theoretical risk of non-specific interactions between antibodies and analytes (largely ruled out here). A potential limitation we see is that smaller batch productions multiplex assay plates less practical, and hence would raise the cost versus the production of larger ones. Nevertheless, with increasing need for an objective multiplex format assays—such as ours—will become overall cheaper than running separate experiments of multiple analytes.

In summary, our tetraplex platform now permits a more objective, faster and more informative interrogation of small volumes of CSF from patients with incurable disorders of the brain. We envision that technological advances such as this one will serve patient stratification, improve the design of clinical trials, allow for disease monitoring, and thus, increase the chance for an urgently needed, positive outcome in future intervention studies.

## Supporting Information

S1 Fig α-Synuclein (A), Aβ42 (B) and Tau protein (C) concentrations measured in either commercially available or validated ELISA assays or multiplex assay are highly correlated. (DOC)

S2 Fig α-Synuclein (A), DJ-1 (B) and Tau protein (C) concentrations measured in singleplex and multiplex assays are highly correlated. (DOC)

S1 Table Raw data for standard curves performed in seven independent experiments. Indicated are mean signal readings and standard deviations. The relatively high standard deviation of Tau protein standard curves may be explained by usage of two different kit lots. This table refers to Fig 1.
(DOC)

S2 Table Raw data for standard curves on individual and pooled standards. Raw data for standard curves performed on individual standards and pooled standards (in duplicate) in a single experiment are indicated. Signal readings for both standard curve formats are highly comparable. This table refers to Fig 2.
(DOC)

S3 Table Raw data of signal readings (S3A Table) and concentrations (in pg/ml; S3B Table) of specific and unspecific interactions in multiplex assays. Indicated are row positions of a 96 well plate, spot with identity of capture antibodies and the respective results. Positions where specific results were expected are highlighted in grey. In most assay wells no unspecific interactions were observed. Only in a few cases unspecific interactions were found (indicated in red in S3B Table). This table refers to Table 2.
(DOC)

S4 Table Raw data of protein concentrations in dilution linearity experiments. Indicated are protein concentrations from CSF samples spiked with very high protein concentrations (left section). Results were then adjusted for the fourth dilution (middle section) and normalized for this dilution step (right section). This table refers to Fig 3.
(DOC)

S5 Table Raw data of protein concentrations in parallelism experiments. Indicated are protein concentrations from serially diluted CSF samples (left section). Results were then adjusted for dilution factors (middle section) and normalized for the fourth dilution step (right section). This table refers to Fig 4.
(DOC)

S6 Table Raw data of protein concentrations from spike recovery experiments. Indicated are protein concentrations in spike solutions used in the experiment as well as protein concentrations measured in CSF samples spiked at different concentrations (left section). Endogenous protein concentrations were subtracted and recovery rates were calculated based on proteins concentrations in spike solutions and spiked CSF samples. This table refers to Fig 5.
(DOC)

S7 Table Raw data of protein concentrations measured in CSF samples from eight neurological control patients and eight patients with Parkinson´s disease in singleplex and multiplex assays. This table refers to Fig 6.
(DOC)

## References

[1] Shi M, Bradner J, Hancock AM, Chung KA, Quinn JF, Peskind ER, et al Cerebrospinal fluid biomarkers for Parkinson disease diagnosis and progression. Ann Neurol. 2010;69: 570–580. 10.1002/ana.22311 PMC3117674 21400565

[2] Abdi F, Quinn JF, Jankovic J, McIntosh M, Leverenz JB, Peskind E, et al Detection of biomarkers with a multiplex quantitative proteomic platform in cerebrospinal fluid of patients with neurodegenerative disorders. J Alzheimers Dis. 2006;9: 293–348. 16914840 10.3233/jad-2006-9309

[3] Ellington AA, Kullo IJ, Bailey KR, Klee GG. Measurement and quality control issues in multiplex protein assays: a case study. Clin Chem. 2009;55: 1092–1099. 10.1373/clinchem.2008.120717 19372187 PMC2965639

[4] Ostendorff HP, Awad A, Braunschweiger KI, Liu Z, Wan Z, Rothschild KJ, et al Multiplexed VeraCode bead-based serological immunoassay for colorectal cancer. J Immunol Methods 2013;400–401: 58–69. 10.1016/j.jim.2013.09.013 PMC3867820 24161315

[5] Hong Z, Shi M, Chung KA, Quinn JF, Peskind ER, Galasko D, et al DJ-1 and alpha-synuclein in human cerebrospinal fluid as biomarkers of Parkinson's disease. Brain 2010;133: 713–726. 10.1093/brain/awq008 20157014 PMC2842513

[6] Mollenhauer B, Locascio JJ, Schulz-Schaeffer W, Sixel-Doring F, Trenkwalder C, Schlossmacher MG. alpha-Synuclein and tau concentrations in cerebrospinal fluid of patients presenting with parkinsonism: a cohort study. Lancet Neurol. 2011;10: 230–240. 10.1016/S1474-4422(11)70014-X 21317042

[7] Blennow K, Hampel H. CSF markers for incipient Alzheimer's disease. Lancet Neurol. 2003;2: 605–613. 10.1016/S1474-4422(03)00530-1 14505582

[8] Dubois B, Feldman HH, Jacova C, Dekosky ST, Barberger-Gateau P, Cummings J, et al Research criteria for the diagnosis of Alzheimer's disease: revising the NINCDS-ADRDA criteria. Lancet Neurol. 2007;6: 734–746. 10.1016/S1474-4422(07)70178-3 17616482

[9] Albert MS, DeKosky ST, Dickson D, Dubois B, Feldman HH, Fox NC, et al The diagnosis of mild cognitive impairment due to Alzheimer's disease: recommendations from the National Institute on Aging-Alzheimer's Association workgroups on diagnostic guidelines for Alzheimer's disease. Alzheimers Dement. 2011;7: 270–279. 10.1016/j.jalz.2011.03.008 21514249 PMC3312027

[10] Schade S, Mollenhauer B. Biomarkers in biological fluids for dementia with Lewy bodies. Alzheimers Res Ther. 2014;6: 72–78. 10.1186/s13195-014-0072-3 eCollection 2014. 25478030 PMC4255553

[11] Schulz-Schaeffer WJ. The synaptic pathology of alpha-synuclein aggregation in dementia with Lewy bodies, Parkinson's disease and Parkinson's disease dementia. Acta Neuropathol. 2010;120: 131–143. 10.1007/s00401-010-0711-0 20563819 PMC2892607

[12] Gouras GK, Tampellini D, Takahashi RH, Capetillo-Zarate E. Intraneuronal beta-amyloid accumulation and synapse pathology in Alzheimer's disease. Acta Neuropathol. 2010;119: 523–541. 10.1007/s00401-010-0679-9 20354705 PMC3183823

[13] Kanemaru K, Kameda N, Yamanouchi H. Decreased CSF amyloid beta42 and normal tau levels in dementia with Lewy bodies. Neurology 2000;54: 1875–1876. 10802808 10.1212/wnl.54.9.1875

[14] Clark CM, Xie S, Chittams J, Ewbank D, Peskind E, Galasko D, et al Cerebrospinal fluid tau and beta-amyloid: how well do these biomarkers reflect autopsy-confirmed dementia diagnoses? Arch Neurol. 2003;60: 1696–1702. 10.1001/archneur.60.12.1696 14676043

[15] Mollenhauer B, Cepek L, Bibl M, Wiltfang J, Schulz-Schaeffer WJ, Ciesielczyk B, et al Tau protein, Abeta42 and S-100B protein in cerebrospinal fluid of patients with dementia with Lewy bodies. Dement Geriatr Cogn Disord. 2005;19: 164–170. 10.1159/000083178 15637452

[16] Aerts MB, Esselink RA, Abdo WF, Bloem BR, Verbeek MM. CSF alpha-synuclein does not differentiate between parkinsonian disorders. Neurobiol Aging 2012;33: 430–433. 10.1016/j.neurobiolaging.2010.12.001 21236518

[17] Mollenhauer B, El-Agnaf OM, Marcus K, Trenkwalder C, Schlossmacher MG. Quantification of alpha-synuclein in cerebrospinal fluid as a biomarker candidate: review of the literature and considerations for future studies. Biomark Med. 2010;4: 683–699. 10.2217/bmm.10.90 20945981

[18] Bastarache JA, Koyama T, Wickersham NE, Ware LB. Validation of a multiplex electrochemiluminescent immunoassay platform in human and mouse samples. J Immunol Methods 2014;408: 13–23. 10.1016/j.jim.2014.04.006 24768796 PMC4120713

[19] Fauvet B, Mbefo MK, Fares MB, Desobry C, Michael S, Ardah MT, et al alpha-Synuclein in Central Nervous System and from Erythrocytes, Mammalian Cells, and Escherichia coli Exists Predominantly as Disordered Monomer. J.Biol.Chem. 2012;287: 15345–64. 10.1074/jbc.M111.318949 22315227 PMC3346117

[20] Kruse N, Schulz-Schaeffer WJ, Schlossmacher MG, Mollenhauer B. Development of electrochemiluminescence-based singleplex and multiplex assays for the quantification of alpha-synuclein and other proteins in cerebrospinal fluid. Methods 2012;56: 514–518. 10.1016/j.ymeth.2012.03.016 22465793

[21] Mollenhauer B, Trautmann E, Otte B, Ng J, Spreer A, Lange P, et al alpha-Synuclein in human cerebrospinal fluid is principally derived from neurons of the central nervous system. J Neural Transm. 2012;119: 739–746. 10.1007/s00702-012-0784-0 22426833 PMC3378837

[22] Pan C, Korff A, Galasko D, Ginghina C, Peskind E, Li G, et al Diagnostic Values of Cerebrospinal Fluid T-Tau and Abeta(4)(2) using Meso Scale Discovery Assays for Alzheimer's Disease. J Alzheimers Dis. 2015;45: 709–719. 10.3233/JAD-143099 25613100 PMC4517668

[23] Lee JW, Devanarayan V, Barrett YC, Weiner R, Allinson J, Fountain S, et al Fit-for-purpose method development and validation for successful biomarker measurement. Pharm Res. 2006;23: 312–328. 10.1007/s11095-005-9045-3 16397743

[24] Food and Drug Administration. Guidance for Industry: Bioanalytical Method Validation, U.S. Department of Health and Human Services. 2001. Available: http://www.fda.gov/downloads/Drugs/…/Guidances/ucm070107.pdf

[25] European Medicine Agency. Guideline on biomedical method validation. 2011. Available: http://www.ema.europa.eu/docs/en_GB/document_library/Scientific_guideline/2011/08/WC500109686.pdf

[26] Mollenhauer B, Cullen V, Kahn I, Krastins B, Outeiro TF, Pepivani I, et al Direct quantification of CSF alpha-synuclein by ELISA and first cross-sectional study in patients with neurodegeneration. Exp Neurol. 2008;213: 315–325. 10.1016/j.expneurol.2008.06.004 18625222

[27] Leviton A, Allred EN, Dammann O, Engelke S, Fichorova RN, Hirtz D, et al Systemic inflammation, intraventricular hemorrhage, and white matter injury. J Child Neurol. 2013;28: 1637–1645. 10.1177/0883073812463068 23112243 PMC4166653

[28] Sharma J, Gray KP, Harshman LC, Evan C, Nakabayashi M, Fichorova R, et al Elevated IL-8, TNF-α, and MCP-1 in men with metastatic prostate cancer starting androgen-deprivation therapy (ADT) are associated with shorter time to castration-resistance and overall survival. Prostate 2014;74: 820–828. 10.1002/pros.22788 24668612

[29] Marchese RD, Puchalski D, Miller P, Antonello J, Hammond O, Green T, et al Optimization and validation of a multiplex, electrochemiluminescence-based detection assay for the quantitation of immunoglobulin G serotype-specific antipneumococcal antibodies in human serum. Clin Vaccine Immunol. 2009;16: 387–396. 10.1128/CVI.00415-08 19158284 PMC2650878

[30] Thway T, Macaraeg C, Calamba D, Patel V, Tsoi J, Ma M, et al Applications of a planar electrochemiluminescence platform to support regulated studies of macromolecules: benefits and limitations in assay range. J Pharm Biomed Anal. 2010;51: 626–632. 10.1016/j.jpba.2009.09.035 19850429

[31] Badiola N, de Oliveira RM, Herrera F, Guardia-Laguarta C, Goncalves SA, Pera M, et al Tau enhances alpha-synuclein aggregation and toxicity in cellular models of synucleinopathy. PLoS One 2011;6: e26609 10.1371/journal.pone.0026609 22039514 PMC3200341

[32] Jensen PH, Hager H, Nielsen MS, Hojrup P, Gliemann J, Jakes R. alpha-synuclein binds to Tau and stimulates the protein kinase A-catalyzed tau phosphorylation of serine residues 262 and 356. J Biol Chem. 1999;274: 25481–25489. 10.1074/jbc.274.36.25481 10464279

[33] Clinton LK, Blurton-Jones M, Myczek K, Trojanowski JQ, LaFerla FM. Synergistic Interactions between Abeta, tau, and alpha-synuclein: acceleration of neuropathology and cognitive decline. J Neurosci. 2010;30: 7281–7289. 10.1523/JNEUROSCI.0490-10.2010 20505094 PMC3308018

[34] Mattsson N, Andreasson U, Persson S, Arai H, Batish SD, Bernardini S, et al The Alzheimer's Association external quality control program for cerebrospinal fluid biomarkers. Alzheimers Dement. 2011;7: 386–395. 10.1016/j.jalz.2011.05.2243 21784349 PMC3710290

[35] Kruse N, Persson S, Bahl JMC, Baldeiras I, Capello E, Bocchio Chiavetto L, et al Validation of a quantitative cerebrospinal fluid alpha-synuclein assay in a European-wide interlaboratory study. Neurobiol Aging 2015;36: 2587–2596. 10.1016/j.neurobiolaging.2015.05.003 26093515

[36] Montine TJ, Shi M, Quinn JF, Peskind ER, Craft S, Ginghina C, et al CSF Abeta(42) and tau in Parkinson's disease with cognitive impairment. Mov Disord. 2010;25: 2682–2685. 10.1002/mds.23287 20818673 PMC2978754

