# Supplementary material for: A First Tetraplex Assay for the Simultaneous Quantification of Total α-Synuclein, Tau, β-Amyloid42 and DJ-1 in Human Cerebrospinal Fluid
Source: PLoS One. 2016 Apr 26;11(4):e0153564. doi: 10.1371/journal.pone.0153564 (PMC4846093; doi:10.1371/journal.pone.0153564)
Supplement: S1 Fig — (DOC) [file pone.0153564.s001.doc]

# Supporting Information

**S1 Fig: aSynuclein (A), Abeta42 (B) and Tau protein (C) concentrations measured in either commercially available or validated ELISA assays or multiplex assay are highly correlated.**

**S1A Fig**

**S1B Figure**

**S1C Figure**
