# Supplementary material for: A First Tetraplex Assay for the Simultaneous Quantification of Total α-Synuclein, Tau, β-Amyloid42 and DJ-1 in Human Cerebrospinal Fluid
Source: PLoS One. 2016 Apr 26;11(4):e0153564. doi: 10.1371/journal.pone.0153564 (PMC4846093; doi:10.1371/journal.pone.0153564)
Supplement: S1 Table — Indicated are mean signal readings and standard deviations. The relatively high standard deviation of Tau protein standard curves may be explained by usage of two different kit lots. This table refers to Fig 1. (DOC) [file pone.0153564.s003.doc]

# Supporting Information

**S1 Table: Raw data for standard curves performed in seven independent experiments.**

| aSynuklein concentration (pg/ml) | Signal mean | SD | Abeta 42 concentration (pg/ml) | Signal mean | SD |
| --- | --- | --- | --- | --- | --- |
| 25000,00 | 1188688 | 307950 | 3000,00 | 664942 | 133674 |
| 6250,00 | 449012 | 114921 | 750,00 | 177667 | 52853 |
| 1562,50 | 108123 | 27569 | 187,50 | 33257 | 12099 |
| 390,63 | 19395 | 4990 | 46,88 | 5072 | 1958 |
| 97,66 | 4323 | 1312 | 11,72 | 1158 | 397 |
| 24,41 | 1158 | 336 | 2,93 | 436 | 99 |
| 6,10 | 450 | 121 | 0,73 | 296 | 45 |
| 0 | 209 | 58 | 0 | 233 | 34 |

| DJ1 concentration (pg/ml) | Signal mean | SD | Tau Protein concentration (pg/ml) | Signal mean | SD |
| --- | --- | --- | --- | --- | --- |
| 25000,00 | 457771 | 59212 | 25000,00 | 482642 | 332484 |
| 6250,00 | 272734 | 29539 | 6250,00 | 115015 | 76888 |
| 1562,50 | 76903 | 8738 | 1562,50 | 26948 | 17824 |
| 390,63 | 15825 | 1808 | 390,63 | 6552 | 4321 |
| 97,66 | 3489 | 540 | 97,66 | 1763 | 968 |
| 24,41 | 1130 | 243 | 24,41 | 607 | 223 |
| 6,10 | 473 | 135 | 6,10 | 306 | 51 |
| 0 | 272 | 124 | 0 | 193 | 64 |

Indicated are mean signal readings and standard deviations. The relatively high standard deviation of Tau protein standard curves may be explained by usage of two different kit lots

This table refers to Fig 1.
