# Supplementary material for: A First Tetraplex Assay for the Simultaneous Quantification of Total α-Synuclein, Tau, β-Amyloid42 and DJ-1 in Human Cerebrospinal Fluid
Source: PLoS One. 2016 Apr 26;11(4):e0153564. doi: 10.1371/journal.pone.0153564 (PMC4846093; doi:10.1371/journal.pone.0153564)
Supplement: S2 Table — Raw data for standard curves performed on individual standards and pooled standards (in duplicate) in a single experiment are indicated. Signal readings for both standard curve formats are highly comparable. This table refers to Fig 2. (DOC) [file pone.0153564.s004.doc]

# Supporting Information

**S2 Table: Raw data for standard curves on individual and pooled standards.**

| aSynuklein concentration (pg/ml) | Signal individual | Signal mean pooled | Abeta 42 concentration (pg/ml) | Signal individual | Signal mean pooled |
| --- | --- | --- | --- | --- | --- |
| 25000,00 | 1141815 | 1059321 | 3000,00 | 590444 | 614495 |
| 6250,00 | 558120 | 639280 | 750,00 | 145783 | 192287 |
| 1562,50 | 158158 | 193634 | 187,50 | 27337 | 36504 |
| 390,63 | 42262 | 43624 | 46,88 | 4562 | 6254 |
| 97,66 | 9027 | 9156 | 11,72 | 1100 | 1424 |
| 24,41 | 2016 | 2335 | 2,93 | 428 | 583 |
| 6,10 | 612 | 703 | 0,73 | 253 | 392 |
| 0 | 104 | 233 | 0 | 184 | 311 |

| DJ1 concentration (pg/ml) | Signal individual | Signal mean pooled | Tau Protein concentration (pg/ml) | Signal individual | Signal mean pooled |
| --- | --- | --- | --- | --- | --- |
| 25000,00 | 480240 | 435428 | 25000,00 | 745220 | 636090 |
| 6250,00 | 242815 | 231672 | 6250,00 | 153108 | 174008 |
| 1562,50 | 58649 | 57589 | 1562,50 | 34541 | 39339 |
| 390,63 | 13388 | 13427 | 390,63 | 9178 | 9909 |
| 97,66 | 3242 | 3302 | 97,66 | 2404 | 2602 |
| 24,41 | 933 | 1157 | 24,41 | 744 | 804 |
| 6,10 | 333 | 611 | 6,10 | 291 | 327 |
| 0 | 122 | 417 | 0 | 122 | 156 |

Raw data for standard curves performed on individual standards and pooled standards (in duplicate) in a single experiment are indicated. Signal readings for both standard curve formats are highly comparable.

This table refers to Fig 2.
