# Supplementary material for: A First Tetraplex Assay for the Simultaneous Quantification of Total α-Synuclein, Tau, β-Amyloid42 and DJ-1 in Human Cerebrospinal Fluid
Source: PLoS One. 2016 Apr 26;11(4):e0153564. doi: 10.1371/journal.pone.0153564 (PMC4846093; doi:10.1371/journal.pone.0153564)
Supplement: S3 Table — Indicated are row positions of a 96 well plate, spot with identity of capture antibodies and the respective results. Positions where specific results were expected are highlighted in grey. In most assay wells no unspecific interactions were observed. Only in a few cases unspecific interactions were found (indicated in red in S3B Table). This table refers to Table 2. (DOC) [file pone.0153564.s005.doc]

# Supporting Information

**S3 Table: Raw data of signal readings (S3A Table) and concentrations (in pg/ml; S3B Table) of specific and unspecific interactions in multiplex assays.**

Supplementary Table 3A

| Row | Spot | Aβ42 standard | Tau standard | DJ-1 standard | aSynuclein standard | pooled standard |  |
| --- | --- | --- | --- | --- | --- | --- | --- |
| A | DJ-1 | 160 | 1099 | 480240 | 160 | 428139 | 442717 |
| A | Tau | 141 | 745220 | 138 | 121 | 647665 | 624514 |
| A | aSyn | 148 | 1564 | 241 | 1141815 | 1045084 | 1073558 |
| A | Abeta | 590444 | 1097 | 225 | 198 | 625411 | 603578 |
| B | DJ-1 | 177 | 359 | 242815 | 151 | 228960 | 234384 |
| B | Tau | 147 | 153108 | 146 | 141 | 176078 | 171937 |
| B | aSyn | 153 | 493 | 208 | 558120 | 614139 | 664420 |
| B | Abeta | 145783 | 497 | 203 | 217 | 195538 | 189035 |
| C | DJ-1 | 160 | 282 | 58649 | 1557 | 57132 | 58046 |
| C | Tau | 127 | 34541 | 145 | 131 | 39314 | 39363 |
| C | aSyn | 140 | 250 | 167 | 158158 | 187879 | 199388 |
| C | Abeta | 27337 | 295 | 199 | 203 | 36311 | 36697 |
| D | DJ-1 | 142 | 250 | 13388 | 123 | 13313 | 13541 |
| D | Tau | 122 | 9178 | 135 | 96 | 10084 | 9733 |
| D | aSyn | 130 | 204 | 126 | 42262 | 43602 | 43646 |
| D | Abeta | 4562 | 239 | 173 | 137 | 6428 | 6080 |
| E | DJ-1 | 138 | 217 | 3242 | 116 | 3030 | 3573 |
| E | Tau | 107 | 2404 | 104 | 98 | 2561 | 2643 |
| E | aSyn | 136 | 162 | 108 | 9027 | 9134 | 9177 |
| E | Abeta | 1100 | 217 | 154 | 144 | 1376 | 1471 |
| F | DJ-1 | 143 | 222 | 933 | 102 | 1195 | 1119 |
| F | Tau | 110 | 744 | 108 | 89 | 809 | 799 |
| F | aSyn | 131 | 172 | 119 | 2016 | 2417 | 2253 |
| F | Abeta | 428 | 219 | 167 | 130 | 608 | 558 |
| G | DJ-1 | 148 | 225 | 333 | 104 | 607 | 614 |
| G | Tau | 114 | 291 | 115 | 100 | 324 | 330 |
| G | aSyn | 111 | 162 | 108 | 612 | 678 | 727 |
| G | Abeta | 253 | 221 | 160 | 134 | 375 | 408 |
| H | DJ-1 | 141 | 227 | 122 | 110 | 408 | 426 |
| H | Tau | 122 | 122 | 107 | 94 | 160 | 152 |
| H | aSyn | 121 | 170 | 106 | 104 | 221 | 245 |
| H | Abeta | 184 | 227 | 168 | 156 | 304 | 318 |
|  |  |  |  |  |  |  |  |

**S3B** Table

| Row | Spot | Aβ42 standard | Tau standard | DJ-1 standard | aSynuclein standard | pooled standard |  |
| --- | --- | --- | --- | --- | --- | --- | --- |
| A | DJ-1 | 0,00 | 24,87 | 29908,68 | 0,00 | 21699,62 | 23647,26 |
| A | Tau | 0,00 | 28907,98 | 0,00 | 0,00 | 25110,22 | 24209,63 |
| A | aSyn | 0,00 | 16,69 | 0,09 | 29874,51 | 20979,35 | 23101,52 |
| A | Abeta | 2765,89 | 8,74 | 0,00 | 0,00 | 3021,32 | 2859,30 |
| B | DJ-1 | 0,00 | 0,00 | 7790,94 | 0,00 | 7177,84 | 7413,87 |
| B | Tau | 0,00 | 5921,94 | 0,00 | 0,00 | 6811,15 | 6650,83 |
| B | aSyn | 0,00 | 3,86 | 0,00 | 5640,37 | 6525,08 | 7417,29 |
| B | Abeta | 610,14 | 2,55 | 0,00 | 0,00 | 773,30 | 798,12 |
| C | DJ-1 | 0,00 | 0,00 | 1552,24 | 40,09 | 1536,32 | 1512,21 |
| C | Tau | 0,00 | 1332,72 | 0,00 | 0,00 | 1517,53 | 1519,42 |
| C | aSyn | 0,00 | 0,27 | 0,00 | 1302,64 | 1551,47 | 1649,69 |
| C | Abeta | 149,04 | 0,00 | 0,00 | 0,00 | 188,01 | 189,65 |
| D | DJ-1 | 0,00 | 0,00 | 373,60 | 0,00 | 371,61 | 377,68 |
| D | Tau | 0,00 | 350,15 | 0,00 | 0,00 | 385,28 | 371,67 |
| D | aSyn | 0,00 | 0,00 | 0,00 | 370,75 | 381,58 | 381,94 |
| D | Abeta | 33,97 | 0,00 | 0,00 | 0,00 | 45,40 | 43,33 |
| E | DJ-1 | 0,00 | 0,00 | 92,28 | 0,00 | 85,92 | 102,13 |
| E | Tau | 0,00 | 87,31 | 0,00 | 0,00 | 93,41 | 96,60 |
| E | aSyn | 0,00 | 0,00 | 0,00 | 90,21 | 91,19 | 91,58 |
| E | Abeta | 8,77 | 0,00 | 0,00 | 0,00 | 11,20 | 12,00 |
| F | DJ-1 | 0,00 | 0,00 | 19,14 | 0,00 | 28,13 | 25,55 |
| F | Tau | 0,00 | 22,79 | 0,00 | 0,00 | 25,32 | 24,93 |
| F | aSyn | 0,00 | 0,00 | 0,00 | 21,68 | 25,98 | 24,23 |
| F | Abeta | 1,62 | 0,00 | 0,00 | 0,00 | 3,87 | 3,29 |
| G | DJ-1 | 0,00 | 0,00 | 0,00 | 0,00 | 7,22 | 7,49 |
| G | Tau | 0,00 | 5,15 | 0,00 | 0,00 | 6,44 | 6,67 |
| G | aSyn | 0,00 | 0,00 | 0,00 | 5,42 | 6,26 | 6,88 |
| G | Abeta | 0,00 | 0,00 | 0,00 | 0,00 | 0,81 | 1,33 |
| H | DJ-1 | 0,00 | 0,00 | 0,00 | 0,00 | 0,00 | 0,00 |
| H | Tau | 0,00 | 0,00 | 0,00 | 0,00 | 0,04 | 0,00 |
| H | aSyn | 0,00 | 0,00 | 0,00 | 0,00 | 0,00 | 0,17 |
| H | Abeta | 0,00 | 0,00 | 0,00 | 0,00 | 0,00 | 0,00 |

Indicated are row positions of a 96 well plate, spot with identity of capture antibodies and the respective results. Positions where specific results were expected are highlighted in grey. In most assay wells no unspecific interactions were observed. Only in a few cases unspecifc interactions were found (indicated in red in S3B Table).

This table refers to Table 2.
