# Supplementary material for: A First Tetraplex Assay for the Simultaneous Quantification of Total α-Synuclein, Tau, β-Amyloid42 and DJ-1 in Human Cerebrospinal Fluid
Source: PLoS One. 2016 Apr 26;11(4):e0153564. doi: 10.1371/journal.pone.0153564 (PMC4846093; doi:10.1371/journal.pone.0153564)
Supplement: S4 Table — Indicated are protein concentrations from CSF samples spiked with very high protein concentrations (left section). Results were then adjusted for the fourth dilution (middle section) and normalized for this dilution step (right section). This table refers to Fig 3. (DOC) [file pone.0153564.s006.doc]

# Supporting Information

**S4 Table: Raw data of protein concentrations in dilution linearity experiments.**

| fold excess aSynuclein | aSyn (pg/ml) | | | adjusted protein concentration (pg/ml) | | | normalized data | | |
| --- | --- | --- | --- | --- | --- | --- | --- | --- | --- |
|  | CSF 1 | CSF 2 | CSF 3 | CSF 1 | CSF 2 | CSF 3 | CSF 1 | CSF 2 | CSF 3 |
| 100 | 2619 | 2759 | 2357 | 327 | 345 | 295 | 94 | 90 | 69 |
| 50 | 1686 | 1626 | 1799 | 422 | 407 | 450 | 121 | 106 | 105 |
| 25 | 900 | 810 | 863 | 450 | 405 | 432 | 129 | 106 | 101 |
| 12,5 | 349 | 382 | 428 | 349 | 382 | 428 | 100 | 100 | 100 |
| 6,25 | 193 | 181 | 201 | 386 | 362 | 402 | 111 | 95 | 94 |
| 3,125 | 90 | 86 | 96 | 360 | 344 | 384 | 103 | 90 | 90 |
| 1,5625 | 46 | 44 | 49 | 368 | 352 | 392 | 105 | 92 | 92 |

| fold excess Abeta42 | Abeta42 (pg/ml) | | | adjusted protein concentration (pg/ml) | | | normalized data | | |
| --- | --- | --- | --- | --- | --- | --- | --- | --- | --- |
|  | CSF 1 | CSF 2 | CSF 3 | CSF 1 | CSF 2 | CSF 3 | CSF 1 | CSF 2 | CSF 3 |
| 25 | 692 | 732 | 609 | 87 | 92 | 76 | 91 | 76 | 62 |
| 12,5 | 478 | 516 | 531 | 120 | 129 | 133 | 126 | 107 | 108 |
| 6,25 | 256 | 256 | 266 | 128 | 128 | 133 | 135 | 106 | 108 |
| 3,125 | 95 | 121 | 123 | 95 | 121 | 123 | 100 | 100 | 100 |
| 1,5625 | 50 | 54 | 56 | 100 | 108 | 112 | 105 | 89 | 91 |
| 0,78125 | 24 | 25 | 28 | 96 | 100 | 112 | 101 | 83 | 91 |
| 0,390625 | 12 | 12 | 13 | 96 | 96 | 104 | 101 | 79 | 85 |

| fold excess DJ1 | DJ1 (pg/ml) | | | adjusted protein concentration (pg/ml) | | | normalized data | | |
| --- | --- | --- | --- | --- | --- | --- | --- | --- | --- |
|  | CSF 1 | CSF 2 | CSF 3 | CSF 1 | CSF 2 | CSF 3 | CSF 1 | CSF 2 | CSF 3 |
| 100 | 18916 | 18073 | 21873 | 2365 | 2259 | 2734 | 37 | 31 | 35 |
| 50 | 18110 | 16520 | 19213 | 4528 | 4130 | 4803 | 70 | 56 | 62 |
| 25 | 13944 | 12302 | 12271 | 6972 | 6151 | 6136 | 108 | 84 | 80 |
| 12,5 | 6442 | 7319 | 7709 | 6442 | 7319 | 7709 | 100 | 100 | 100 |
| 6,25 | 3824 | 3502 | 3841 | 7648 | 7004 | 7682 | 119 | 96 | 100 |
| 3,125 | 1798 | 1693 | 1977 | 7192 | 6772 | 7908 | 112 | 93 | 103 |
| 1,5625 | 889 | 810 | 922 | 7112 | 6480 | 7376 | 110 | 89 | 96 |

| fold excess Tau | Tau Protein (pg/ml) | | | adjusted protein concentration (pg/ml) | | | normalized data | | |
| --- | --- | --- | --- | --- | --- | --- | --- | --- | --- |
|  | CSF 1 | CSF 2 | CSF 3 | CSF 1 | CSF 2 | CSF 3 | CSF 1 | CSF 2 | CSF 3 |
| 100 | 8372 | 6010 | 4968 | 1047 | 751 | 621 | 127 | 88 | 70 |
| 50 | 4817 | 3029 | 2879 | 1204 | 757 | 720 | 146 | 89 | 82 |
| 25 | 1809 | 1602 | 1611 | 905 | 801 | 806 | 110 | 94 | 91 |
| 12,5 | 824 | 854 | 883 | 824 | 854 | 883 | 100 | 100 | 100 |
| 6,25 | 443 | 446 | 471 | 886 | 892 | 942 | 108 | 104 | 107 |
| 3,125 | 245 | 247 | 279 | 980 | 988 | 1116 | 119 | 116 | 126 |
| 1,5625 | 139 | 137 | 147 | 1112 | 1096 | 1176 | 135 | 128 | 133 |

Indicated are protein concentrations from CSF samples spiked with very high protein concentrations (left section). Results were then adjusted for the fourth dilution (middle section) and normalized for this dilution step (right section).

This table refers to Fig 3.
