# Supplementary material for: A First Tetraplex Assay for the Simultaneous Quantification of Total α-Synuclein, Tau, β-Amyloid42 and DJ-1 in Human Cerebrospinal Fluid
Source: PLoS One. 2016 Apr 26;11(4):e0153564. doi: 10.1371/journal.pone.0153564 (PMC4846093; doi:10.1371/journal.pone.0153564)
Supplement: S5 Table — Indicated are protein concentrations from serially diluted CSF samples (left section). Results were then adjusted for dilution factors (middle section) and normalized for the fourth dilution step (right section). This table refers to Fig 4. (DOC) [file pone.0153564.s007.doc]

# Supporting Information

**S5 Table: Raw data of protein concentrations in parallelism experiments.**

| aSyn | aSyn (pg/ml) pooled CSF | | | | adjusted protein concentration (pg/ml) | | | | normalized data | | | |
| --- | --- | --- | --- | --- | --- | --- | --- | --- | --- | --- | --- | --- |
|  | pooled CSF | CSF 1 | CSF 2 | CSF 3 | pooled CSF | CSF 1 | CSF 2 | CSF 3 | pooled CSF | CSF 1 | CSF 2 | CSF 3 |
| neat | 77 | 110 | 66 | 66 | 77 | 110 | 66 | 66 | 19 | 31 | 18 | 27 |
| 1:2 | 114 | 86 | 84 | 66 | 228 | 172 | 168 | 132 | 57 | 48 | 47 | 53 |
| 1:4 | 85 | 74 | 75 | 49 | 340 | 296 | 300 | 196 | 85 | 82 | 83 | 79 |
| 1:8 | 50 | 45 | 45 | 31 | 400 | 360 | 360 | 248 | 100 | 100 | 100 | 100 |

| Ab42 | Abeta42 (pg/ml) | | | | adjusted protein concentration | | | | normalized data | | | |
| --- | --- | --- | --- | --- | --- | --- | --- | --- | --- | --- | --- | --- |
|  | pooled CSF | CSF 1 | CSF 2 | CSF 3 | pooled CSF | CSF 1 | CSF 2 | CSF 3 | pooled CSF | CSF 1 | CSF 2 | CSF 3 |
| neat | 41 | 73 | 38 | 43 | 41 | 73 | 38 | 43 | 9 | 18 | 9 | 17 |
| 1:2 | 114 | 72 | 77 | 47 | 228 | 144 | 154 | 94 | 51 | 35 | 35 | 37 |
| 1:4 | 90 | 80 | 94 | 55 | 360 | 320 | 376 | 220 | 80 | 78 | 85 | 86 |
| 1:8 | 56 | 51 | 55 | 32 | 448 | 408 | 440 | 256 | 100 | 100 | 100 | 100 |

| DJ1 | DJ1 (pg/ml) | | | | adjusted protein concentration | | | | normalized data | | | |
| --- | --- | --- | --- | --- | --- | --- | --- | --- | --- | --- | --- | --- |
|  | pooled CSF | CSF 1 | CSF 2 | CSF 3 | pooled CSF | CSF 1 | CSF 2 | CSF 3 | pooled CSF | CSF 1 | CSF 2 | CSF 3 |
| neat | 490 | 581 | 547 | 371 | 490 | 581 | 547 | 371 | 63 | 69 | 58 | 83 |
| 1:2 | 329 | 360 | 389 | 217 | 658 | 720 | 778 | 434 | 84 | 85 | 83 | 97 |
| 1:4 | 204 | 217 | 236 | 117 | 816 | 868 | 944 | 468 | 104 | 102 | 101 | 104 |
| 1:8 | 98 | 106 | 117 | 56 | 784 | 848 | 936 | 448 | 100 | 100 | 100 | 100 |

| Tau Protein | Tau Protein (pg/ml) | | | | adjusted protein concentration | | | | normalized data | | | |
| --- | --- | --- | --- | --- | --- | --- | --- | --- | --- | --- | --- | --- |
|  | pooled CSF | CSF 1 | CSF 2 | CSF 3 | pooled CSF | CSF 1 | CSF 2 | CSF 3 | pooled CSF | CSF 1 | CSF 2 | CSF 3 |
| neat | 150 | 296 | 255 | 170 | 150 | 296 | 255 | 170 | 51 | 59 | 47 | 63 |
| 1:2 | 124 | 196 | 190 | 116 | 248 | 392 | 380 | 232 | 84 | 78 | 70 | 85 |
| 1:4 | 74 | 118 | 117 | 68 | 296 | 472 | 468 | 272 | 100 | 94 | 86 | 100 |
| 1:8 | 37 | 63 | 68 | 34 | 296 | 504 | 544 | 272 | 100 | 100 | 100 | 100 |

Indicated are protein concentrations from serially diluted CSF samples (left section). Results were then adjusted for dilution factors (middle section) and normalized for the fourth dilution step (right section).

This table refers to Fig 4.
