# Supplementary material for: A First Tetraplex Assay for the Simultaneous Quantification of Total α-Synuclein, Tau, β-Amyloid42 and DJ-1 in Human Cerebrospinal Fluid
Source: PLoS One. 2016 Apr 26;11(4):e0153564. doi: 10.1371/journal.pone.0153564 (PMC4846093; doi:10.1371/journal.pone.0153564)
Supplement: S6 Table — Indicated are protein concentrations in spike solutions used in the experiment as well as protein concentrations measured in CSF samples spiked at different concentrations (left section). Endogenous protein concentrations were subtracted and recovery rates were calculated based on proteins concentrations in spike solutions and spiked CSF samples. This table refers to Fig 5. (DOC) [file pone.0153564.s008.doc]

# Supporting Information

**S6 Table: Raw data of protein concentrations from spike recovery experiments.**

|  | aSyn spike (pg/ml) | CSF + spike | | | | endogenous protein concentration subtracted | | | | % recovery | | | |
| --- | --- | --- | --- | --- | --- | --- | --- | --- | --- | --- | --- | --- | --- |
|  |  | CSF 1 | CSF 2 | CSF 3 | CSF 4 | CSF 1 | CSF 2 | CSF 3 | CSF 4 | CSF 1 | CSF 2 | CSF 3 | CSF 4 |
| spike high | 2388 | 2840 | 1171 | 1633 | 1146 | 2085 | 1133 | 1583 | 1100 | 87 | 47 | 66 | 46 |
| spike mid | 1052 | 1785 | 541 | 791 | 647 | 1030 | 503 | 741 | 601 | 98 | 48 | 70 | 57 |
| spike low | 535 | 1194 | 292 | 416 | 302 | 439 | 254 | 366 | 256 | 82 | 47 | 68 | 48 |
| CSF neat |  | 755 | 38 | 50 | 46 |  |  |  |  |  |  |  |  |

|  | Ab42 spike (pg/ml) | CSF + spike | | | | endogenous protein concentration subtracted | | | | % recovery | | | |
| --- | --- | --- | --- | --- | --- | --- | --- | --- | --- | --- | --- | --- | --- |
|  |  | CSF 1 | CSF 2 | CSF 3 | CSF 4 | CSF 1 | CSF 2 | CSF 3 | CSF 4 | CSF 1 | CSF 2 | CSF 3 | CSF 4 |
| spike high | 765 | 574 | 365 | 551 | 365 | 544 | 318 | 511 | 328 | 71 | 42 | 67 | 43 |
| spike mid | 365 | 288 | 194 | 302 | 234 | 258 | 147 | 262 | 197 | 71 | 40 | 72 | 54 |
| spike low | 202 | 146 | 128 | 178 | 121 | 116 | 81 | 138 | 84 | 57 | 40 | 68 | 42 |
| CSF neat |  | 30 | 47 | 40 | 37 |  |  |  |  |  |  |  |  |

|  | DJ1 spike (pg/ml) | CSF + spike | | | | endogenous protein concentration subtracted | | | | % recovery | | | |
| --- | --- | --- | --- | --- | --- | --- | --- | --- | --- | --- | --- | --- | --- |
|  |  | CSF 1 | CSF 2 | CSF 3 | CSF 4 | CSF 1 | CSF 2 | CSF 3 | CSF 4 | CSF 1 | CSF 2 | CSF 3 | CSF 4 |
| spike high | 2458 | 2784 | 2296 | 2513 | 2408 | 2416 | 2139 | 2298 | 2182 | 98 | 87 | 93 | 89 |
| spike mid | 1112 | 1485 | 1266 | 1386 | 1377 | 1117 | 1109 | 1171 | 1151 | 100 | 100 | 105 | 104 |
| spike low | 549 | 912 | 682 | 770 | 794 | 544 | 525 | 555 | 568 | 99 | 96 | 101 | 103 |
| CSF neat |  | 368 | 157 | 215 | 226 |  |  |  |  |  |  |  |  |

|  | Tau Protein spike (pg/ml) | CSF + spike | | | | endogenous protein concentration subtracted | | | | % recovery | | | |
| --- | --- | --- | --- | --- | --- | --- | --- | --- | --- | --- | --- | --- | --- |
|  |  | CSF 1 | CSF 2 | CSF 3 | CSF 4 | CSF 1 | CSF 2 | CSF 3 | CSF 4 | CSF 1 | CSF 2 | CSF 3 | CSF 4 |
| spike high | 762 | 888 | 661 | 959 | 842 | 849 | 638 | 892 | 774 | 111 | 84 | 117 | 102 |
| spike mid | 428 | 474 | 353 | 500 | 467 | 435 | 330 | 433 | 399 | 102 | 77 | 101 | 93 |
| spike low | 228 | 248 | 202 | 283 | 280 | 209 | 179 | 216 | 212 | 92 | 79 | 95 | 93 |
| CSF neat |  | 39 | 23 | 67 | 68 |  |  |  |  |  |  |  |  |

Indicated are protein concentrations in spike solutions used in the experiment as well as protein concentrations measured in CSF samples spiked at different concentrations (left section). Endogenous protein concentrations were subtracted and recovery rates were calculated based on proteins concentrations in spike solutions and spiked CSF samples.

This table refers to Fig 5.
