# Supplementary material for: A First Tetraplex Assay for the Simultaneous Quantification of Total α-Synuclein, Tau, β-Amyloid42 and DJ-1 in Human Cerebrospinal Fluid
Source: PLoS One. 2016 Apr 26;11(4):e0153564. doi: 10.1371/journal.pone.0153564 (PMC4846093; doi:10.1371/journal.pone.0153564)
Supplement: S7 Table — This table refers to Fig 6. (DOC) [file pone.0153564.s009.doc]

# Supporting Information

**S7 Table: Raw data of protein concentrations measured in CSF samples from eight neurological control patients and eight patients with Parkinson´s disease in singleplex and multiplex assays.**

|  | aSynuklein (pg/ml) (singleplex) | aSynuklein (pg/ml) (multiplex) | DJ1 (pg/ml) (singleplex) | DJ1 (pg/ml) (multiplex) | Tau Protein (pg/ml) (singleplex) | Tau Protein (pg/ml) (multiplex) |  | Abeta42 (pg/ml) (multiplex) |
| --- | --- | --- | --- | --- | --- | --- | --- | --- |
| NC 1 | 165 | 86 | 220 | 919 | 73 | 47 |  | 127 |
| NC 2 | 119 | 71 | 229 | 855 | 28 | 26 |  | 90 |
| NC 3 | 143 | 72 | 259 | 915 | 61 | 61 |  | 64 |
| NC 4 | 155 | 75 | 212 | 885 | 49 | 58 |  | 109 |
| NC 5 | 222 | 140 | 379 | 1359 | 67 | 81 |  | 56 |
| NC 6 | 299 | 88 | 347 | 1088 | 77 | 69 |  | 99 |
| NC 7 | 155 | 64 | 266 | 1035 | 52 | 64 |  | 65 |
| NC 8 | 147 | 74 | 239 | 889 | 33 | 43 |  | 48 |
|  |  |  |  |  |  |  |  |  |
| PD 1 | 180 | 104 | 264 | 1027 | 29 | 35 |  | 95 |
| PD 2 | 132 | 66 | 197 | 738 | 35 | 43 |  | 62 |
| PD 3 | 85 | 33 | 161 | 583 | 22 | 20 |  | 35 |
| PD 4 | 125 | 67 | 210 | 889 | 28 | 32 |  | 89 |
| PD 5 | 177 | 82 | 210 | 779 | 28 | 35 |  | 50 |
| PD 6 | 211 | 110 | 285 | 1131 | 41 | 52 |  | 167 |
| PD 7 | 144 | 64 | 200 | 1022 | 35 | 41 |  | 64 |
| PD 8 | 116 | 62 | 143 | 696 | 18 | 31 |  | 73 |

This table refers to Fig 6.
